# A new classification network for diagnosing Alzheimer's disease in class-imbalance MRI datasets

**DOI:** 10.3389/fnins.2022.807085

**Published:** 2022-08-25

**Authors:** Ziyang Chen, Zhuowei Wang, Meng Zhao, Qin Zhao, Xuehu Liang, Jiajian Li, Xiaoyu Song

**Affiliations:** ^1^School of Computers, Guangdong University of Technology, Guangzhou, China; ^2^School of Computer Science and Engineering, Tianjin University of Technology, Tianjin, China; ^3^Department of Electrical and Computer Engineering, Portland State University, Portland, OR, United States

**Keywords:** Alzheimer's disease diagnosis, class-imbalance problem, classification network, lightweight blocks, global contextual information, gradient density

## Abstract

Automatic identification of Alzheimer's Disease (AD) through magnetic resonance imaging (MRI) data can effectively assist to doctors diagnose and treat Alzheimer's. Current methods improve the accuracy of AD recognition, but they are insufficient to address the challenge of small interclass and large intraclass differences. Some studies attempt to embed patch-level structure in neural networks which enhance pathologic details, but the enormous size and time complexity render these methods unfavorable. Furthermore, several self-attention mechanisms fail to provide contextual information to represent discriminative regions, which limits the performance of these classifiers. In addition, the current loss function is adversely affected by outliers of class imbalance and may fall into local optimal values. Therefore, we propose a 3D Residual RepVGG Attention network (ResRepANet) stacked with several lightweight blocks to identify the MRI of brain disease, which can also trade off accuracy and flexibility. Specifically, we propose a Non-local Context Spatial Attention block (NCSA) and embed it in our proposed ResRepANet, which aggregates global contextual information in spatial features to improve semantic relevance in discriminative regions. In addition, in order to reduce the influence of outliers, we propose a Gradient Density Multiple-weighting Mechanism (GDMM) to automatically adjust the weights of each MRI image *via* a normalizing gradient norm. Experiments are conducted on datasets from the Alzheimer's Disease Neuroimaging Initiative (ADNI) and Australian Imaging, Biomarker and Lifestyle Flagship Study of Aging (AIBL). Experiments on both datasets show that the accuracy, sensitivity, specificity, and Area Under the Curve are consistently better than for state-of-the-art methods.

## 1. Introduction

Alzheimer's disease (AD) is an incurable neurodegenerative disorder with the loss of tissues and death of nerve cells throughout the brain. Clinically, the typical features of AD are memory impairment, executive dysfunction, and aphasia, for example. As of 2018, patients with AD are generally over the age of 65 years, with a prevalence of approximately 6%, while the death ratio due to AD has increased by 145% (Alam et al., 2017; Wood, 2018). Therefore, the early detection of AD is significant for patients and may help to alleviate the risk and morbidity of AD. Many studies have reported on auxiliary algorithms based on Machine Learning (ML) methods to construct a system for the early detection of AD and to recognize differences between AD and Normal Cognitive function (NC).

Traditionally, ML algorithms are able to achieve automatic classification tasks by learning complex and subtle biomarkers. For instance, Moore et al. (2019) applied the Support Vector Machine (SVM) classifier to identify patients with AD by constructing a hyperplane to maximize the margin and distinguish different features between AD and NC. In addition, Ashburner and Friston (2000) proposed a Random Forest (RF) tree to predict the probability of NC to AD at different time intervals. These algorithms can initially distinguish between MRI of AD and NC, but the semantic features extracted by these encoders, such as SVM or RF trees, are weak representations. This is an obstacle to improving the accurate recognition of patients with AD. Therefore, further studies have attempted to explore each scan for informative features to improve the semantic features of the lesion, including voxel-level, region-level, and patch-level. Specially, the voxel-level methods (Khvostikov et al., 2018) used a *t*-test to select more informative features of representing AD. However, the regular ML algorithms mentioned are readily subject to overfitting, increasing the challenge of AD recognition tasks in small interclass and large intraclass differences.

Following trends in neural networks in medical image analysis, some researchers have attempted to combine types of neural networks with traditional ML methods to alleviate the problem of overfitting (Billones et al., 2016; Rocca et al., 2018; Yee et al., 2020). For instance, Rocca et al. (2018) employed a two-dimensional (2D) neural network with a patch-level feature selection strategy to divide the whole MRI into several patches, improving feature discrimination to alleviate overfitting to some extent. However, MRIs commonly consist of a large number of patches, which slows down the detection speed. Therefore, Yee et al. (2020) constructed a three-dimensional (3D) subject-level network with a dilated module instead of an extended patch-level feature selection strategy, to achieve high-level feature extraction at high speed. Other studies have explored different kinds of networks expanded by a subject-level network to apply to AD classification tasks, including DemNet, VoxCNN, residual plain networks, and dense-like networks (Korolev et al., 2017; Stoyanov, 2018; Cui and Liu, 2019). Some studies have found that the full connection layer is largely ineffective in extracting all the spatial information from the output of the network encoder. Therefore, researchers have attempted alternative methods, using extra module-like bidirectional long short-term memory (Bi-LSTM) and bidirectional gated recurrent units (Bi-GRUs) (Korolev et al., 2017; Stoyanov, 2018; Cui and Liu, 2019) into fully connection layers to jointly learn spatial and longitudinal features. Above all, current networks can achieve a more robust performance than ML algorithms in recognizing MRIs with clear lesion features, but they find it difficult to further distinguish features between patients with AD and older patients with NC. It is a challenge for the current networks to improve the accurate identification of AD because the brain features of the elderly are similar to some AD features. Therefore, some studies have embedded an attention mechanism (Jin et al., 2019; Tong et al., 2019; Alahmari, 2020; Cheng et al., 2020; Chen et al., 2021) to improve relevant features and allow older patients with NC to be more easily distinguished from patients with AD. For example, Cheng et al. (2020) devised a Fully-Convolutional Attention Network (FCANet) to segment chest X-rays effectively. This network aggregated contextual biomarker information from long-range and short-range distances. Meanwhile, Jin et al. (2019) and Chen et al. (2021) proposed an Attention- ResNet to incorporate spatial-awareness into each feature position and enhance feature representation.

Overall, the attention networks described above are designed to integrate fine-grained spatial information to focus on more relevant features. However, the performance of the attention networks is easily influenced by the data of the imbalance category in practice. In particular, the number of patients with AD is less than the number of NC subjects, which reduces the capacity of AD feature extraction. Therefore, how to accurately extract AD-related features to adapt to this category imbalance has become key to improving model performance. Some studies (Ouyang et al., 2020; Zhao et al., 2020) have attempted to design an effective loss function to alleviate the problem of category imbalance. For example, the focal dice loss was proposed by Zhao et al. (2020) to reduce the contribution from easy samples, enabling the model to focus on hard samples. In addition, Ouyang et al. (2020) proposed a gradient-based online trainable loss function instead of the focal dice loss to consider the gradient of each MRI weight hard sample, which can efficiently achieve a neural network with a robust attention mechanism in typical conditions.

Generally speaking, the method can improve discriminative features from different angles such as network structure, different attention mechanisms, and loss function. They have improved the performance of the models from the perspective of feature discrimination and class-imbalance to alleviate the challenge of small interclass and large intraclass differences. However, the challenges are still remain. First, the common classification networks fail to resolve the contradiction between speed and accuracy in recognizing the MRI of Alzhemier's disease. Second, in the clinical setting, some local abnormal regions (Alexiou et al., 2019) are influenced by patient factors such as age and sex, for example. These factors decrease the semantic relevance of the abnormal regions, making it difficult for the current attention mechanism to recognize patients with AD. Finally, the current loss function of category imbalance affected by outliers is associated with an increased risk of trapping in the local optimum. Therefore, in order to overcome these challenges and release the potential of deep neural networks, we extended the 2D RepVGG training model (Ding et al., 2021) to a 3D variant, and devised a novel network that makes a tradeoff between speed (structure of sufficiently light weight) and accuracy (ability to extract semantic features). Thereafter, we embedded a novel self-attention module to integrate a global context into the semantic features, to alleviate the limitation of spatial information. The outputs of our proposed network are fed into the newly class-imbalance function we propose to balance the weights of each MRI, including outliers *via* normalized gradient density inspired by the gradient-based method proposed by Li et al. (2019). The contributions of this article can be summarized as follows:

1. In order to be tradeoff the speed and accuracy, a 3D Residual RepVGG Attention Network (ResRepANet) is proposed. The ResRepANet consists of several lightweight Blocks, called RepBlocks, and each of them can extract AD features precisely.
2. To enhance the semantic relevance of discriminative regions and decrease the influence of external factors such as age and gender, we embedded a Non-local Context Spatial Attention (NCSA) block in the proposed network. Specifically, this block aggregates context and spatial features to enhance the semantic of lesion features caused by brain atrophy.
3. We reveal a new strategy, called Gradient Density Multi-weighting Mechanism (GDMM), to alleviate the influence of outliers when the model is trained directly on class imbalanced data. We applied the statistical gradient histogram to understand the distribution of difficult samples (outliers) and easy samples in each batch which can balance the weights between outliers and easy samples dynamically.

The remainder of this article is organized as follows. Section 2 presents a description of the proposed method, and Section 3 summarizes our experimental results and their analysis. Finally, Section 4 presents the conclusions of this study.

## 2. Methods

**Figure 1:**
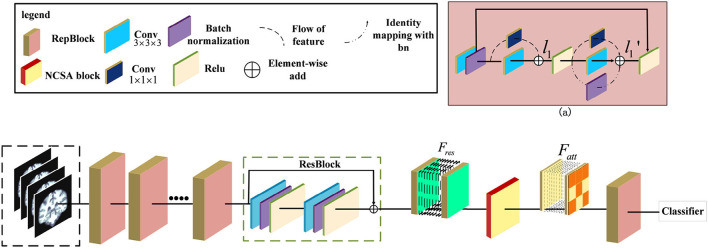
Illustration of the pipeline for Alzheimer's Disease classification. The ResRepANet consists of several ResRepBlocks, ResBlock, and a Non-local Context Spatial Attention (NCSA) block. (a) Structure of ResRepBlock, where the feature *l*_1_ extracted from the convolution module with kernel size 3 × 3 × 3 and 1 × 1 × 1 as well as the feature $l_{1}^{\prime }$ extracted from two convolution modules and identity mapping.

In this section, we provide the details of our proposed 3D Residual RepVGG Attention Network (ResRepANet) in terms of network architecture, NCSA, and GDMM. The architecture of ResRepANet is shown in Figure 1, which aims to explore the discriminative features of AD classification under class-imbalance. First, the high-level features are encoded by several ResRepBlocks and ResBlock which will be detailed in Section 2.1. Then, the NCSA block is exploited to extract the context feature and spatial feature of AD, as described in Section 2.2. Finally, in Section 2.3, the GDMM is proposed to balance the gradient of the model for each MRI and alleviate the influence of outliers. In addition, details of the implementation of ResRepANet are presented in Section 2.4.

### 2.1. The architecture of 3D residual RepVGG attention network

Some researchers attempted that the complicated multi-branch structures, such as ResNet and Inception module, improve the feature discriminative of lesions, but slow down the inference times (Ding et al., 2021). Specifically, these structures with cross-layer connections reduce memory utilization. This situation also exists in the field of medical image recognition, where current deep learning networks hardly balanced the speed and accuracy. We observe that the RepVGG can achieve the high performance of classification tasks in ImageNet whatever inference time or Identification accuracy. Specifically, these structures with cross-layer connections reduce memory utilization. This situation also exists in the field of medical image recognition, where current deep learning networks hardly balanced the speed and accuracy. Besides, this network applied the same style of VGG and embedded the re-parameterization method to decrease the inference time. Therefore, inspired by this design, we first construct the basic module based on the ResNet 3D with pretrained parameters. Then, we extended the 2D RepVGG training model to a 3D variant and devised a novel network, called ResRepANet, to perform the MRI classification task in Alzheimer's disease. As can be seen in Figure 1, the feature map X' is extracted by the convolutional block with kernel size 3 × 3 × 3 and the convolutional block with kernel size 1 × 1 × 1 and generated two types of feature maps. Subsequently, these features map are fused to the feature map *l*_1_ by performing the operation of element-wise add. Besides, the *l*_1_ feature map is extracted by three blocks, including two types of convolutional block and batch normalization layers, and outputs three feature tensors with the same shape, respectively. Finally, these features map are merged to the feature map $l_{1}^{\prime }$ by performing the operation of element-wise add. Here are the details of constructing the ResRepANet. First, MRIs are fed into ResRepBlocks to extract the features of different resolutions. Specifically, the structure of ResRepBlock consists solely of the convolution block with kernel sizes 3 × 3 × 3 and 1 × 1 × 1 in order to maintain the high speed of inference. The purpose of designing this ResRepBlock is that the block can extract features with different resolutions to generate different scales of semantic information. This approach is suitable for the classification of complex features such as the texture of brain contours. More formally, the MRI of inputs is defined as *X* ∈ ℝ^*h*×*w*×*d*^ while the convolution modules *f*_3×3×3_ and *f*_1×1× 1_ represent the kernel sizes of 3 × 3 × 3 and 1 × 1 × 1, respectively. The feature map *X*′ is mapped by the *f*_3×3×3_ and batch normalization module *f*_*bn*_, as shown in Figure 1.


(1) $$\begin{array}{l} X^{\prime }=f_{bn}\left(f_{3\times 3\times 3}\left(X\right)\right) \end{array}$$

Next, the feature map *X*′ is fed into two branches with *f*_3×3×3_ and *f*_1×1×1_ respectively, generating the output features *l*_1_ . The output feature *l*_1_ is defined as follows and is aggregated by the feature map with filtering by *f*_3×3×3_ and *f*_1×1×1_.


(2) $$\begin{array}{l} l_{1}=f_{3\times 3\times 3}\left(X^{\prime }\right)+f_{1\times 1\times 1}\left(X^{\prime }\right) \end{array}$$

The feature map is passed through the activation function Relu to improve generalization and then fed into the parallel structure with three branches. Next, the output feature map $l_{1}^{\prime }$ is generated by the output of the three branches, and the feature mapped identity is defined from *l*_1_, with the summation of element-wise as follows.


(3) $$\begin{array}{l} l_{1}^{\prime }=f_{3\times 3\times 3}\left(l_{1}\right)+f_{1\times 1\times 1}\left(l_{1}\right)+f_{bn}\left(l_{1}\right) \end{array}$$

where *f*_*bn*_(*l*_1_) is a function of batch normalization.

Second, the NCSA block is subject to the feature *F*_*res*_ , which is the feature map $l_{1}^{\prime }$ smoothed by the ResBlock. To aggregate the context feature into the spatial feature, the NCSA block is exploited to multiply the attention map with the context features to enhance the relevance of each feature. Finally, the output of the last ResRepBlock is input into the global average pooling and used to predict the category probability, as shown in Figure 1. The main reason for inserting a layer capable of global average pooling is to progressively reduce the size of the spatial representation. We next introduce the NCSA block and GDMM in detail.

### 2.2. The structure of non-local context spatial attention block

**Figure 2:**
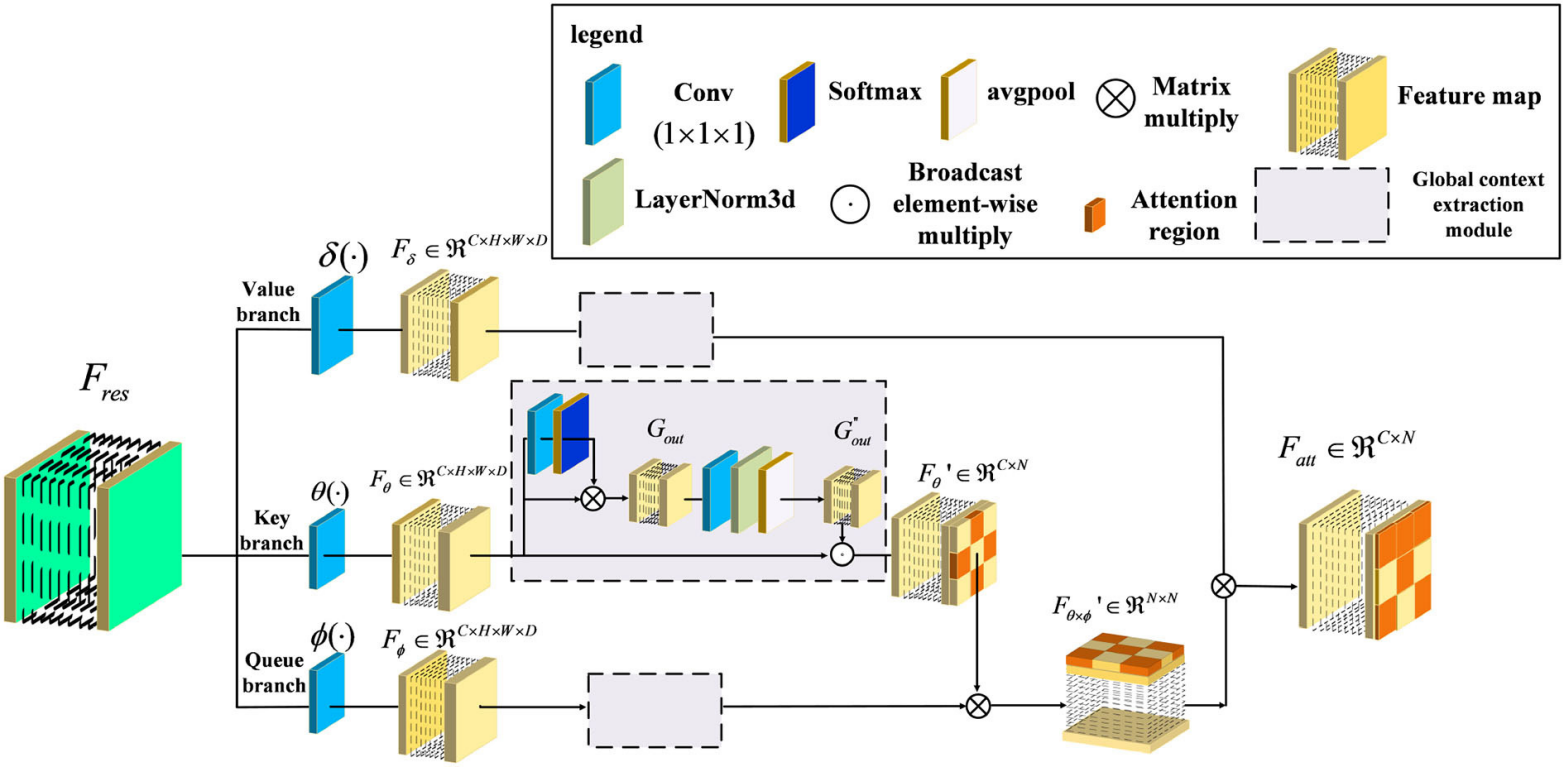
Illustration of Non-local Context Spatial Attention (NCSA) block. The NCSA block consists of the global context extraction module which aggregated the global context to the spatial feature. Here, ϕ(·), θ(·), and δ(·) represent a query branch filter, a key branch filter, and a value branch filter, respectively.

In general, the above variants of improvement strategies have been derived for the attention mechanism, broadly speaking for the channel domain, the spatial domain, the hybrid domain, and self-attention mechanisms. They can achieve robust performance in 2D medical image recognition based on enhancing channel and spatial features. However, the performance of the above attention structure is inhibited when extracting the regions with low lesion recognition. Specifically, some lesion regions, such as the lobe or hippocampus, appear to have slight atrophy under the influence of Alzheimer's disease. Therefore, we proposed a novel attention structure, called Non-local Context Spatial Attention block (NCSA block), to improve the feature semantics of lesion regions. In this block, we first construct the self-attention framework to generate basic feature association space. Meanwhile, the global context extraction module is embedded into the above framework to expand the feature semantics of lesions, which further enhanced the extent of region atrophy. Different from other combined attention structures, the global context module is embedded in each branch of the self-attention framework directly. The above module can integrate the context feature to each region with feature discriminative by the operation of the self-attention mechanism. The details are shown in Figure 2. Specifically, three filters are constructed to encode feature information at the beginning of the NCSA block including a query branch filter ϕ(·), a key branch filter θ(·), and a value branch filter δ(·). The feature map *F*_*res*_, as the input of the NCSA block, extracted by ResBlock is fed into three filters and generated the feature embedding *F*_ϕ_, *F*_δ_, and *F*_θ_, respectively. Here, ϕ(·), θ(·), and δ(·) are all the convolution blocks with kernel size *f*_1×1×1_.

Furthermore, in order to strengthen context information of feature embedding, we employed a global context extraction module in each branch. As can be seen in Figure 2, we define ${G_{out}}^{\prime \prime }$ as the global context feature. Here, we choose the feature map from the Key branch as an example. Note that, the feature map *F*_θ_ is mapped by the convolutional block with the kernel size 1 × 1 × 1, and the receptive field has not changed. Therefore, the feature map *F*_θ_ also contains the global position features. The task of the global context extraction module is to activate these position features and explore the context features. Here, we design two steps to explore the global context feature from the feature map *F*_θ_. The first step is to enhance the position feature from the spatial domain and the second step is to improve the context feature in the channel domain. First of all, the feature map *F*_θ_ is mapped by the convolutional block with kernel size 1 × 1 × 1 and generates the feature map with one channel. After that, we transform this feature with the softmax function into a weight tensor. Subsequently, the *G*_*out*_ is generated by performing a matrix multiply operation with the original feature *F*_θ_ and the weight tensor and finishes the first step of position enhancement in the spatial domain. Second, the *G*_*out*_ is smoothed by the convolutional block with kernel size 1 × 1 × 1. Here, the output channel of the feature is the same as the input channel. Then, the layer normalization module is proposed to normalize the feature ${G_{out}}^{\prime }$ in the channel dimension. After that, the adaptive average pooling is embedded in the layer normalization module to integrate all of the spatial features and generate the global context feature ${G_{out}}^{\prime \prime }$. Finally, the $F_{\theta }^{\prime }$ is calculated by the *F*_θ_ to perform the operation of broadcast element-wise multiplied with the ${G_{out}}^{\prime \prime }$ and finish the second step of context enhancement in the channel domain. The formula to complete this process is as follows:


(4) $$\begin{array}{l} G_{out}\left(x_{i}\right)=F_{\theta }⊗\overset{N}{\underset{i=1}{\sum }}\beta \left(G_{i}\right) \end{array}$$

where *i* is an index of the output position *N* at which the response of position feature *G*_*i*_ is to be computed. The global attention weighting function β(*G*_*i*_) can be defined as:


(5) $$\begin{array}{l} \beta \left(G_{i}\right)={\text{e}}^{G_{i}}∕\overset{N}{\underset{m=1}{\sum }}{\text{e}}^{G_{m}} \end{array}$$

Second, we performed layer normalization to smooth the feature of different channels and the output feature map $G_{out}^{\prime }$, denoted as follows:


(6) $$\begin{array}{l} G_{out}^{\prime }=f_{1\times 1\times 1}\left\{LN\left[f_{1\times 1\times 1}\left(G_{out}\right)\right]\right\} \end{array}$$

Here, *LN* denotes the layer normalization module which can normalize each channel of features. In order to transform the position relevance between each feature to context information, we utilized global average pooling *P*_*avg*_ to aggregate the position feature map $G_{out}^{\prime }$ and mapping into the context feature map $G_{out}^{\prime \prime }$. Here, the shape of the context attention map $G_{out}^{\prime \prime }$ is squeezed from four dimensions of the feature map (*C*,*h*,*w*,*d*) to two dimensions of the feature map (*C*, *h*×*w*×*d*). Next, in order to integrate the context information into the feature embedding and generate a more informative feature map, we combined the context feature $G_{out}^{\prime \prime }$ with the feature embedding *F*_θ_ by the operation of broadcast element-wise multiplication, and generated the feature map $F_{\theta }^{\prime }$. Here, we have finished the second step of context enhancement in the channel domain. The formula to complete this process is as follows:


(7) $$\begin{array}{l} G_{out}^{\prime \prime }=P_{avg}\left(G_{out}^{\prime }\right) \end{array}$$

(8) $$\begin{array}{l} F_{\theta }^{\prime }=G_{out}^{\prime \prime }⊙F_{\theta } \end{array}$$

Furthermore, the feature map $F_{\theta }^{\prime }$ with context feature is used to explore relevance with the feature map *F*_ϕ_ generated from a queue filter ϕ(·) and generated the feature map of related position $F_{\theta \times \phi }^{\prime }$ as follows:


(9) $$\begin{array}{l} F_{\theta \times \phi }^{\prime }=F_{\phi }⊗\left(G_{out}^{\prime \prime }⊙F_{\theta }\right) \end{array}$$

Here, this feature map can represent the similarity of each feature, which can help the model to locate discriminative regions. Finally, the output feature map $F_{att}\in {ℜ}^{N\times C}$ is considered the whole embedding space of position from $F_{\theta \times \phi }^{\prime }$ to locate the lesion regions based on the discriminative regions. The formula to complete this process in view of Equations (4–9) is as follows:


(10) $$\begin{array}{l} F_{att}=\frac{1}{C\left(x\right)}\underset{\forall j}{\sum }F_{\theta \times \phi }^{\prime }\left(x_{i},x_{i}\right)F_{\delta }\left(x_{j}\right) \end{array}$$

where *x* is the feature value of each feature map and *j* is the index of each position in *F*_δ_, while *C*(*x*) represents the response factor from each feature value *x*. In summary, the NCSA block is constructed from an embedding space to strengthen the semantic relevance of each feature map. Each feature map in this space can be explored using long-range dependence and expanded context information, which improves representative lesion regions in the MRI.

### 2.3. The design of gradient density multi-weighting mechanism

The common loss function of class-imbalance is to adjust the gradient by setting learnable hyperparameters. However, the model is forcefully focused on the gradient variation of outliers and trapped into a local optimum. Therefore, we propose the GDMM to alleviate the influence of outliers inspired by the gradient-based function. First, we collected gradients of each MRI from the model as well as data on the gradient histogram and divided the value of the histogram into 10 intervals. Second, the number of each interval was calculated as the gradient norm and generated to a region of the gradient norm. Third, the gradient density was computed by the histogram of the region of gradient norm and divided into ‘easy' and ‘hard', and we performed normalization corresponding to the histogram. Finally, we utilized the gradient density to reweight each MRI to balance the gradient contribution between difficult (outlier) and easy samples.

As is well known, the direction of model convergence is decided by the extent of changed error between the value predicted by the model and the ground-truth. Here, we first defined the absolute error *E*_*total*_ between the output of the network ${\hat{y}}_{i}$ and the ground-truth *y*_*i*_ as the formula:


(11) $$\begin{array}{l} E_{total}\text{=}S\left({\hat{y}}_{i}\right)-y_{i} \end{array}$$

where *S*(·) is the sigmoid activation function. In particular, this activation function can squeeze the interval of output into the range from zero to one. Next, we calculated the gradient to measure how easy it is for the function to converge. We defined *grad* to represent the error changes with respect to the value of the weight:


(12) $$\begin{array}{l} grad=\frac{\partial \left(E_{total}\right)}{\partial y_{i}}=\frac{\partial \left(S\left({\hat{y}}_{i}\right)-y_{i}\right)}{\partial y_{i}} \end{array}$$

Furthermore, we can analyze the gradient values to create histograms that give an indication of the training situation. We refer to such histograms as ‘gradient norms'. These gradient norms are utilized to explore the data distribution to metric the current situation of training. In particular, the hard samples were presented with higher gradient norms than the easy samples. If the value of gradient norms is maintained at high values, it means that the model is falling into local optimum which eventually leads to under-fitting. This situation is caused by the class-imbalance problem in which the minor class of sample features affects the sample distribution. In order to further measure the degree of difficulty based on the gradient norm, inspired by the white balance algorithm, we then introduced the function *GD*(*grad*_*N*_) to represent the gradient density of each gradient norm, which can be expressed in the form:


(13) $$\begin{array}{l} GD\left(grad_{N}\right)=\frac{1}{\xi \left(grad\right)}\overset{N}{\underset{n=1}{\sum }}\psi \left(grad_{n},grad\right) \end{array}$$

Here, *grad*_*n*_ is the gradient norm of the n-th example. The function ψ(·, ·) is an index function that is inferred the probability of the predicted value belonging to the gradient norm. The above function ψ(·, ·) can be expressed as follows:


(14) $$\begin{array}{l} \psi \left(grad_{n},grad\right)\text{=}\{\begin{array}{l} 1\text{ if }grad-\frac{\varepsilon }{2}≼grad_{n}≼grad+\frac{\varepsilon }{2}\\ 0\text{ otherwise} \end{array} \end{array}$$

Besides, the ξ(*grad*) yields the interval size of the gradient norm which can be used to calculate the ratio of the gradient norm to the overall gradient norm in the specified interval.


(15) $$\begin{array}{l} \xi \left(grad\right)=\min\left(grad+\frac{\varepsilon }{2},1\right)-\max\left(grad-\frac{\varepsilon }{2},0\right) \end{array}$$

where ε = 1*e*−6. Here, the gradient density can measure the intensity of gradient norms belonging to different intervals. Next, the harmonizing weights *W*_*N*_ are further calculated based on the gradient density, which can be balanced the gradient norm:


(16) $$\begin{array}{l} W_{N}=\frac{N}{GD\left(grad_{N}\right)} \end{array}$$

where *N* is the total number of examples. This parameter can then be incorporated into the classification loss function. Here, the harmonizing parameter *W*_*N*_ is similar to the learning rate of the loss function and can enhance the rate at which the model converges by considering the gradient density.

Finally, we utilized maximum entropy *F*_*max*_ in our proposed GDMM, as this loss can connect the most informative gradient based on informative entropy to adjust the direction of converging. This function keeps a stable sensitivity of harmonizing weights *W*_*N*_ which can avoid the influence of outliers. Here, the process of GDMM can be represented as the loss function *F*_*soft*_ with embedded harmonizing parameters as the formula:


(17) $$\begin{array}{l} F_{soft}=-W_{N}*F_{max} \end{array}$$

Specifically, the maximum entropy loss function, *F*_*max*_, can formula as follows:


(18) $$\begin{array}{l} F_{max}=\overset{N}{\underset{i=1}{\sum }}\hat{y_{i}}*\log{\left(1+e^{-y_{i}}\right)}^{-1}+\left(1-\hat{y_{i}}\right)*\log\left(\frac{e^{-y_{i}}}{1+e^{-y_{i}}}\right) \end{array}$$

Here, $\hat{y_{i}}$ represents the *i*-th prediction output of our model while *y*_*i*_ is the ground-truth label of the classification task.

In summary, the GDMM is optimized by computing harmonizing weights and weighting each batch of training samples based on gradient density. More importantly, the model can dynamically adjust itself by weighting multiple batches, which can balance the sensitivity of the category features between outliers and easy samples. This mechanism can help the model find the best convergence direction without applying oversampling.

### 2.4. Implementation

Our proposed method is an improvement on ResNet18, whose structure is shown in Table 1. First, ResRepBlock-1 is able to receive the MRI data and extract features from them (*via* convolution) into a feature space with 48 channels. In each block, we set the strides to be equal to two in order to extract different resolutions of the feature map. Second, we implemented four ResRepBlocks to extract different scales of dense features. Finally, the global average pooling is employed followed by a fully connected layer to aggregate the feature map from ResRepBlock-4, which can align the shape to fully connected layers. Then, the optimized hyperparameters of the model, including learning rate and weight decay parameters, were tested on the held-out Alzheimer's Disease Neuroimaging Initiative (ADNI) test dataset. To further verify the robustness and generalizability of our model, we also evaluated our model in the internal dataset (Australian Imaging, Biomarker, and Lifestyle Flagship Study of Aging; AIBL). In the training stage, all experiments reported were carried out with an 8-core 16-thread AMD Ryzen 7 3700x CPU and 11GB GTX 2080 Ti GPU. The deep-learning framework adopted was PyTorch ≥ 1.3.1 equipped with the Torchvision ≥0.4.2 software package.

**Table 1:** Architectural specification of the ResRepANet.

**Block**	**Num blocks**	**Input channels**	**Output channels**	**Output size**
ResRepBlock-1	1	48	48	61*73*61
ResRepBlock-2	1	48	48	31*37*31
ResRepBlock-3	1	48	96	16*19*16
ResRepBlock-4	1	96	192	8*10*8
ResBlock3D	4	192	192	4*5*4
NCSA Block	1	192	192	4*5*4
ResRepBlock-5	1	192	384	2*3*2
GAP	1	384	384	1*1*1
Linear	1	384	2	2

## 3. Experiments

In this section, the effectiveness of our method is investigated by carrying out several experiments. First, we describe the datasets that are implemented (Section 3.1) and introduce evaluation metrics (Section 3.2). Second, the analysis of comparative experiments is introduced in detail (Section 3.3). Third, the results of ablation experiments are presented (Section 3.4). Finally, the analysis of model performance is presented *via* visualizing the feature map and heatmap (Section 3.5).

### 3.1. Datasets

(1) *Data selection*: Two datasets (i.e., ADNI and AIBL) were derived from the Alzheimer's Disease Neuroimaging Initiative database (http://adni.loni.usc.edu) and the Australian Imaging, Biomarker, and Lifestyle Flagship Study of Aging database (https://aibl.csiro.au). These datasets consist of T1-weighted MR brain scans that have not been preprocessed, and which are the type of image produced by most MR scanners. Since the main objective of the study was to classify the MRI of patients with AD and CN, only baseline subjects were taken into consideration. In particular, the AIBL was collected at two centers (40% of subjects from Perth in Western Australia, 60% from Melbourne, Victoria), which differs from the ADNI. We consider that different sample distributions from different regions better reflect the true generalizable situation, and selected AIBL as the test set. Therefore, accordingly, the data that met the following criteria were selected. Here, we first selected the ADNI-1, ADNI-2 Initial Visit Dataset, and ADNI-3 Initial Visit Dataset as the main datasets and the AIBL dataset as the test dataset. Next, in order to address redundant subjects, such as those with different modalities or with diagnosis records for different visits, we selected the subjects with only a T1 modality sample who were diagnosed as having AD dementia at the baseline timepoint and maintained this for follow-up. The two datasets (i.e., ADNI and AIBL) were all applied to evaluate the performance and generalizability of our ResRepANet. The allocation ratio between the training and validation set based on ADNI is chosen to be 9:1. Specifically, the validation set was used to tune the hyperparameters, and the weights were learned during the training process.

(2) *Data pre-processing*: The selected data were then preprocessed and underwent smoothing. Specifically, the spatial normalization was done to warp the image into Montreal Neurological Institute (MNI) space and assigned it to the same resolution. The intensity normalization was then performed by dividing each voxel intensity by the global average value. Thereafter, images were further smoothed by a Gaussian kernel with a full width at half maximum of 8 mm. All procedures were implemented with SPM12 (Zhang et al., 2011). After pre-processing, MRI scans were checked manually in order to remove those subjects failed in the processing procedure. In total, 1,251 baseline MRI images from ADNI constituted the experimental dataset, among which 419 subjects had AD and 832 had NC. Demographic and clinical data of subjects is provided in Table 2, in which MMSE stands for the Mini-Mental State Examination. In addition, 531 baseline MRI images from AIBL constituted the test dataset, among which 79 subjects had AD and 451 had NC. In summary, the imbalance ratio between AD and NC in the test part of ADNI was 1:3, compared with 1.7:10 for AIBL.

**Table 2:** Demographic data of subjects included in the study.

**Dataset**	**Category**	**Gender**	**Age(years)**	**MMSE**
ADNI	AD	234/185	76.6 ± 7.4	21.2 ± 4.5
	CN	352/480	77.3 ± 5.3	29.1 ± 1.3
AIBL	AD	30/49	73.4 ± 7.8	20.5 ± 5.7
	CN	196/255	72.8 ± 6.6	28.7 ± 1.2

### 3.2. Experimental Settings

Our proposed method was verified on the AD classification (AD vs. NC) task. We applied four metrics to evaluate the classification performance, including accuracy (ACC), sensitivity (SEN), specificity (SPE), and area under the receiver operating characteristic curve (AUC). Specifically, the ACC means the ratio of the number of samples correctly predicted by the model to the overall sample size. Besides, AUC is calculated on all possible pairs of true positive rate and false positive rate by changing the thresholds performed on the prediction results from our trained network. We measure the model performance by increasing the values of these indicators (ACC, SEN, SPE, and AUC). Higher values indicate better model performance. We set the number of training epochs to 300 and the batch size to 8. Specifically, the initial learning rate is set to 1e-4 and the speed of gradient descent is controlled *via* a warmup policy. Meanwhile, the weight decay of 1e-8 and the momentum of 0.9 are implemented in the SGD optimizer.

### 3.3. Comparison with previous studies

In this section, we first compare the performance of our proposed network with ML algorithms, including a conventional method base on Region of Interest (ROI) (Liu et al., 2012), voxel-level morphometry (VBM) (Ashburner and Friston, 2000), and a patch-level method (PLM) (Penny et al., 2007). Next, we considered the state-of-the-arts to illustrate the effectiveness of our method in the deep learning field. In order to further verify the generality of our method, we further evaluated our method and its competing methods on AIBL. To ensure the performance of our method and its competing methods can compare in a fair manner, all experimental results were obtained in the same experimental environments and same numbers of subjects.

First, the results of the AD classification are shown in Table 3 including our method and its competing ML methods on the test set from ADNI. For example, the patch-level methods (i.e., PLM) (Penny et al., 2007) all outperformed the voxel-level and ROI-level methods (i.e., VBM; Ashburner and Friston, 2000 and ROI; Liu et al., 2012). A possible reason is that the patch-level feature representation can capture more informative features. After that, our method reached better results on all four metrics (i.e., ACC = 0.892, SEN = 0.903, SPE = 0.900, and AUC = 0.940) in AD classification. Compared with the conventional patch-level method (i.e., PLM), our method achieved considerably better results for AD diagnosis. This may be because the feature map generated by our proposed method contains higher level semantic information than the other methods.

**Table 3:** Comparison of our method with previous machine learning studies (AD progression detection based on the ADNI dataset).

**Method**	**ACC**	**SEN**	**SPE**	**AUC**
ROI (Liu et al., 2012)	0.804	0.718	0.888	0.852
VBM (Ashburner and Friston, 2000)	0.816	0.756	0.875	0.883
PLM (Penny et al., 2007)	0.825	0.765	0.850	0.825
Ours	0.892	f0.903	0.900	0.940

Second, the performances on AD classification achieved by our method and the deep learning methods on the test set from ADNI are shown in Table 4. On one hand, Yee et al. (2020), Esmaeilzadeh et al. (2018), and Khvostikov et al. (2018) adopted 3D CNN structure with an accuracy per case of 90.1, 91.7, and 90.5% for AD classification, respectively. Among them, the model from Esmaeilzadeh et al. (2018) produced slightly better results than the other two. In contrast, our proposed method achieved an accuracy 0.5% higher than the best results using the above methods. From the above results, it can be shown that the capacity of the backbone network to encode features determines the upper limit of classification accuracy. Specifically, this structure of our proposed ResRepBlock is more suitable for feature extraction in Alzheimer's disease to improve feature discrimination. Besides, Jin et al. (2019) and Korolev et al. (2017) proposed Attention-ResNet to improve feature discrimination in order to compensate for its capacity for feature encoding. As a result, the accuracy of the method from Jin et al. (2019) is 7.1% higher than that of Korolev et al. (2017), while the sensitivity is 5.3% higher than Korolev et al. (2017). This demonstrates that an attention mechanism can strengthen some relevant information of the feature map to a certain extent, thereby improving the accuracy of the models. Our proposed method outperformed the accuracy of Jin et al. (2019) and Korolev et al. (2017) by about 1.7 and 8.8%, respectively. Although the current attention mechanism (Korolev et al., 2017; Jin et al., 2019) can highlight some relevant information on a feature map, it cannot locate more informative regions. In contrast, the global context module embedded into our NCSA block was intended to integrate the semantic information into each feature map, this enables our model to be located the more informative regions. On the other hand, the performance of Bi-GRU (Cui et al., 2019) is better than that of FSBi-LSTM (Feng et al., 2019), as can be seen in Table 4. As it is affected by category imbalance, the discriminative capacity of Bi-GRU is less than that of our model, so our accuracy is 5.3% higher than that of Cui et al. (2019). Specifically, FSBi-LSTM extracts all the spatial information from the feature maps instead of the fully connected layer which received feature maps with 200 channels *via* a linear transformation. In contrast, Bi-GRU contained an update and reset the gates jointly to learn spatial and longitudinal features and train the disease classifier. Therefore, a reasonable structure of the classifier plays an important role in the accuracy of AD identification. Our proposed method with GDMM function can achieve 4.3% higher accuracy than Cui et al. (2019) and a sensitivity 90.3% higher than Cui et al. (2019), not only because of the reasonable structure of the classifier but also because of the efficient loss function to improve the robustness of the model.

**Table 4:** Comparison of our method with previous deep-learning studies (AD progression detection based on the ADNI dataset).

**Method**	**ACC**	**SEN**	**SPE**	**AUC**
Yee et al. (2020)	0.804	0.803	0.823	0.889
Khvostikov et al. (2018)	0.822	0.821	0.837	0.885
Korolev et al. (2017)	0.804	0.803	0.824	0.876
Liu et al. (2018)	0.804	0.803	0.838	0.899
Cui et al. (2019)	0.839	0.839	0.842	0.899
Jin et al. (2019)	0.875	0.874	0.887	0.885
Esmaeilzadeh et al. (2018)	0.839	0.862	0.850	0.937
Feng et al. (2019)	0.785	0.765	0.799	0.857
Ours	0.892	0.903	0.900	0.940

Finally, to verify the generalizability of our method, we further utilized an independent AIBL dataset to evaluate our method and the competing methods, trained on the ADNI dataset. Observing the results of Tables 5, 6, we can see that our proposed method generally outperforms the other competing ML methods (i.e., ROI, VBM, PLM) in most metrics in both AD-related diagnosis tasks. For example, it achieves an ACC of 0.922 for the AD progression detection task on the AIBL dataset using the model trained on the ADNI dataset, which is better than VBM (0.808), ROI (0.793), and PLM (0.839). The main reason could be that using the discriminative features learned by the NCSA block that can distinguish the patient with AD in total AIBL. Noted that, the SEN of our proposed method achieves 0.855 lower than the PLM. On one hand, the PLM uses a block-level hierarchical extraction algorithm for each block region. This model allows its local feature representation ability more robust. In particular, the model considers that the subject may have AD for some samples with some subtle atrophy features by utilizing patch-level algorithms. These patch-level algorithms can choose several discriminative patches to further find out the difference between the normal entropy and lesioned entropy. The model with higher specificity can help doctors to distinguish the subject whether is normal elder or not. On the other hand, the effect of gender mismatch between training and testing datasets. Noted that All of the methods (i.e., ROI, VBM, PLM, Our methods) are trained in ADNI with a male to female ratio of 4:5, and then tested on the test set AIBL with a male to female ratio of 3:4. The overall distribution of lesion features also changed when the male to female ratio changed. Our proposed method performed relatively consistently in terms of global feature extraction capability but was weaker than the PLM in terms of local features. Additionally, our method still outperformed (Esmaeilzadeh et al., 2018; Jin et al., 2019; Yee et al., 2020) on the AIBL dataset, with 2.1, 0.9, and 0.5% improvement on accuracy for AD classification, respectively. These comparative experiments indicate that our method achieves the best AD classification results on the AIBL dataset, surpassing the results from the state-of-the-arts (SOTA). In conclusion, our proposed method can be adaptive to the ADNI dataset and the AIBL dataset, achieving a more stable performance than its competing methods.

**Table 5:** Comparison of our method with previous machine learning studies (AD progression detection based on the AIBL dataset).

**Method**	**ACC**	**SEN**	**SPE**	**AUC**
ROI (Liu et al., 2012)	0.793	0.519	0.863	0.796
VBM (Ashburner and Friston, 2000)	0.808	0.582	0.866	0.817
PLM (Penny et al., 2007)	0.839	0.722	0.870	0.846
Ours	0.922	0.829	0.855	0.889

**Table 6:** Comparison of our method with previous deep-learning studies (AD progression detection based on the AIBL dataset).

**Method**	**ACC**	**SEN**	**SPE**	**AUC**
Yee et al. (2020)	0.901	0.754	0.826	0.903
Khvostikov et al. (2018)	0.905	0.720	0.874	0.763
Korolev et al. (2017)	0.896	0.756	0.806	0.896
Liu et al. (2018)	0.867	0.718	0.739	0.752
Cui et al. (2019)	0.879	0.662	0.789	0.771
Jin et al. (2019)	0.913	0.781	0.850	0.773
Esmaeilzadeh et al. (2018)	0.917	0.778	0.859	0.868
Feng et al. (2019)	0.905	0.824	0.811	0.764
Ours	0.922	0.829	0.855	0.889

### 3.4. Ablation Study

To evaluate the effectiveness of the ResRepANet embedded in our study, we further compared the proposed methods with their direct counterparts; i.e., the model with only ResRepBlock, the model only with the self-attention module, the model only with the global context extraction module, and the model with all modules. We evaluated these four methods on AD-related diagnosis tasks, with results reported in Table 7. Here, we designed our backbone inspired by ResNet18 and we compare the performance between our backbone and the backbone embedded with the ResRepBlock. First, the performance of ResRepBlock can be seen in the first and second columns in Table 7. The backbone embedded with the ResRepBlock achieved metrics of the accuracy of 0.857, which is 1.8% higher than the result of the backbone without any extra module. It is for this reason that the dense features generated by ResRepBlock are essential for improving feature discriminative. Then, the backbone model combined with the self-attention framework gives more accurate results (by 3.9%) compared with the backbone combined with the global context extraction module. Moreover, our final model is able to achieve an accuracy of 88.4% and an AUC of 93.4%. Clearly, the current attention module is barely adaptive to situations that are close in terms of intraclass and far away in terms of interclass, with the extraction of the global context or semantic feature only. In contrast, our final model allows the positional relationship between pixels and the semantic feature to be extracted simultaneously, which is key to discovery of the discriminative features of the feature map. The feature generated by our final model can be represented by the regions where lesions (atrophy, deformation, etc.) appear. In conclusion, two components are both needed; it is essential that the performance of the attention module is strengthened as much as possible to enable it to extract the necessary semantic information. The best combination of methods is clearly the backbone of NCSA.

**Table 7:** Quantitative evaluation of ablation analysis for different attention structures in the ADNI dataset.

**Structure**	**ACC**	**SEN**	**SPE**	**AUC**
Backbone	0.839	0.838	0.839	0.903
Backbone+R	0.857	0.856	0.858	0.924
Backbone+R+S	0.875	0.874	0.879	0.928
Backbone+R+G	0.839	0.838	0.842	0.913
Backbone+R+NCSA	0.884	0.889	0.893	0.934

R reported as RepResBlock.

S reported as self-attention module.

G reported as global context module.

**Figure 3:**
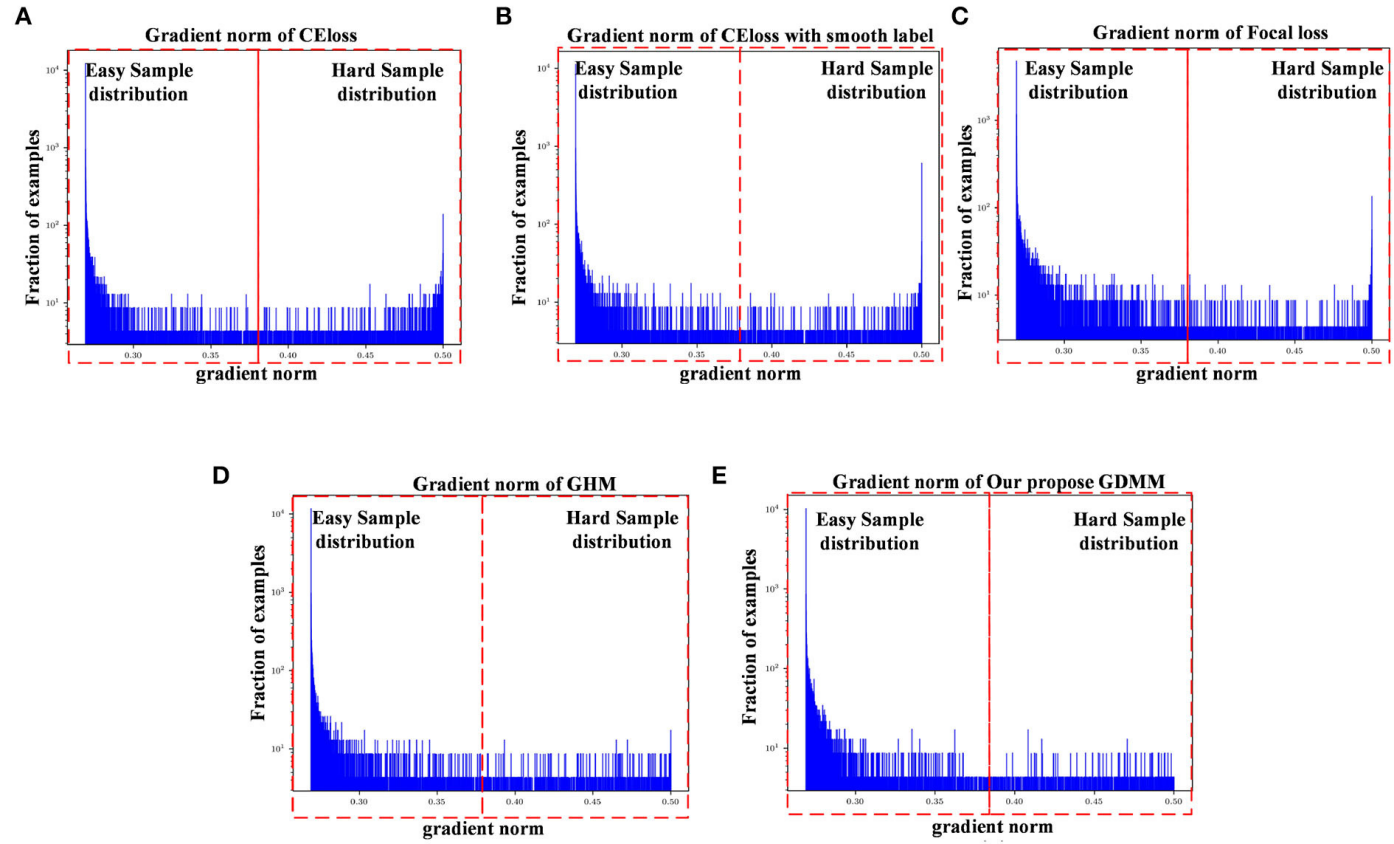
Gradient density corresponding to different loss functions: **(A)** CE loss function, **(B)** CE loss function with label smoothing, **(C)** Focal loss function, **(D)** GHM, and **(E)** Our GDMM. Here, the x-axis represents the predicted value of the gradient norm from the model output, while the y-axis is the number of the grad value set, which is the subtraction between the predicted value and label value that can assess the sample difficulty level. We set two intervals to divide difficulty level, as 0–0.375 (easy sample region) and 0.375–0.5 (hard sample region) in our experiments, with 0.375 as the median value in the predicted value sets.

In order to evaluate the model performance under category imbalance, we compared five different loss functions (CE loss, focal loss, GHM, CE loss with label smoothing, and our GDMM) to investigate the effectiveness of the various strategies. As can be seen in Table 8, our loss function (GDMM) yielded an accuracy of 89.2%, sensitivity of 90.3%, and AUC value of 94%. In contrast, the CE loss function with label smoothing only achieved an accuracy of 82.14% and an AUC of 90.8%. These results imply that our strategy can be adapted well to this imbalanced dataset. On one hand, our proposed GDMM applied a gradient-based strategy to normalize each batch of gradient density which can alleviate the influence of outliers instead of focal loss, while paying greater attention to outliers. On the other hand, considering the challenge of small interclass and large intraclass differences, we embedded maximum entropy into GDMM to further improve the performance of the classifier. In order to show the situation of model convergence, we visualized the gradient norms from these loss functions, giving the results shown in Figure 3.

**Table 8:** Quantitative evaluation of ablation analysis for different loss functions in the ADNI dataset.

**Method**	**ACC**	**SEN**	**SPE**	**AUC**
CE with label smooth	0.821	0.821	0.828	0.908
Focal loss	0.821	0.821	0.836	0.919
GHM	0.839	0.839	0.839	0.913
CE loss	0.884	0.889	0.893	0.934
GDMM	0.892	0.903	0.900	0.940

The ease of convergence of the model is determined by measuring the distribution of the gradient norm of the predicted values. Specifically, the larger the prediction value, the larger the current gradient and the larger the value of the gradient norm. We estimate how well the current model is trained for all samples by counting the gradient values for all samples simultaneously and counting the histogram of these gradient values. If the majority of the samples reflect relatively small gradient values, the model has learned most of the sample features. Specifically, first, we map all the gradient values to 0–1 by sigmoid. Second, we count the gradient norm of all the gradient values at the last epoch. Finally, we classify the difficulty region bound as 0.375, which is the midpoint of the overall gradient norm range (according to the statistics, the overall gradient norm has a maximum value of 0.5). Here, as can be seen in Figure 3, the x-axis represents the gradient norm, while the y-axis (with a log-scale) is the histogram belonging to the gradient norm. The red outlined boxes are the represented location of the specific regions of the sample distributions; the interval from 0 to 0.375 corresponds to the region containing samples that are easy to classify and the interval from 0.375 to 0.5 corresponds to the samples that are hard to classify. Gradient density is generated by the distribution of a gradient norm between the easy region and the hard region. In this case, we can observe the situation of model performance, in which the model can be adapted or not to a hard sample feature by the histograms of gradient density between different loss functions. Compared with the loss function of CE, loss with smooth label and GDMM in Figures 3B,E, we can see that the gradient density in the hard sample region is generally smaller than those in the CE with label smoothing histogram. It can be seen that the gradient norm of each sample is normalized by GDMM, which can balance the weight effectively to decrease the attention on outliers to some extent. Samples with a large gradient density will be reduced, and the weight of samples with a lower density will be increased, such that the various types of samples have a more balanced contribution to the update of model parameters. In summary, our proposed GDMM can reduce the difficulty of model convergence based on adjusting the gradient density dynamically.

We also compared the inference time between our proposed method and its competing methods to fully demonstrate the efficiency of our proposed method, as shown in Table 9. As a result, under the same platform of NVIDIA GTX 1080TI containing a single GPU, Jin et al. (2019) required 176 milliseconds (mms) to infer an unseen testing subject while Korolev et al. (2017) required 183 mms. This is because the methods of Jin et al. (2019) and Korolev (Korolev et al., 2017) adapt the residual structure, which slows down the inference speed. Similarly, the method of Khvostikov (Khvostikov et al., 2018) utilized a two-stream structure with a large scale convolution module, which is the main reason that it is the slowest of all methods. The methods of Feng et al. (2019) and Cui et al. (2019) reached an inference time of more than 200 ms due to the complex structures involved. Our proposed method can achieve a time of 151 mms, which is faster than the other methods. This is because the structure of our proposed method consists of only convolution with kernel size 3 × 3 × 3 embedded into our proposed ResRepBlock, based on a re-parameterization strategy that can achieve a better inference speed while maintaining stable accuracy.

**Table 9:** Comparison of inference time between our proposed methods and other state-of-the-art methods (AD progression detection based on the ADNI dataset).

**Method**	**ACC**	**Times(ms)**
Yee et al. (2020)	0.804	180
Khvostikov et al. (2018)	0.822	230
Korolev et al. (2017)	0.804	183
Liu et al. (2018)	0.804	205
Cui et al. (2019)	0.839	221
Jin et al. (2019)	0.875	176
Esmaeilzadeh et al. (2018)	0.839	171
Feng et al. (2019)	0.785	219
Ours	0.892	151

### 3.5. Visualization

The potential of clinical translation is important to computer-aided diagnosis. One of the keys to the clinical diagnosis of AD is to observe morphological changes in the brain (i.e., to find abnormal atrophy areas of the brain). As an auxiliary diagnostic approach, our proposed method can automatically identify possible pathological locations in the whole MR images, allowing doctors to find the regions of interest for easy diagnosis. In other words, our method can identify subject-specific discriminative pathological locations, including relative discriminative regions in global images and discriminative micro-structures in local patches.

**Figure 4:**
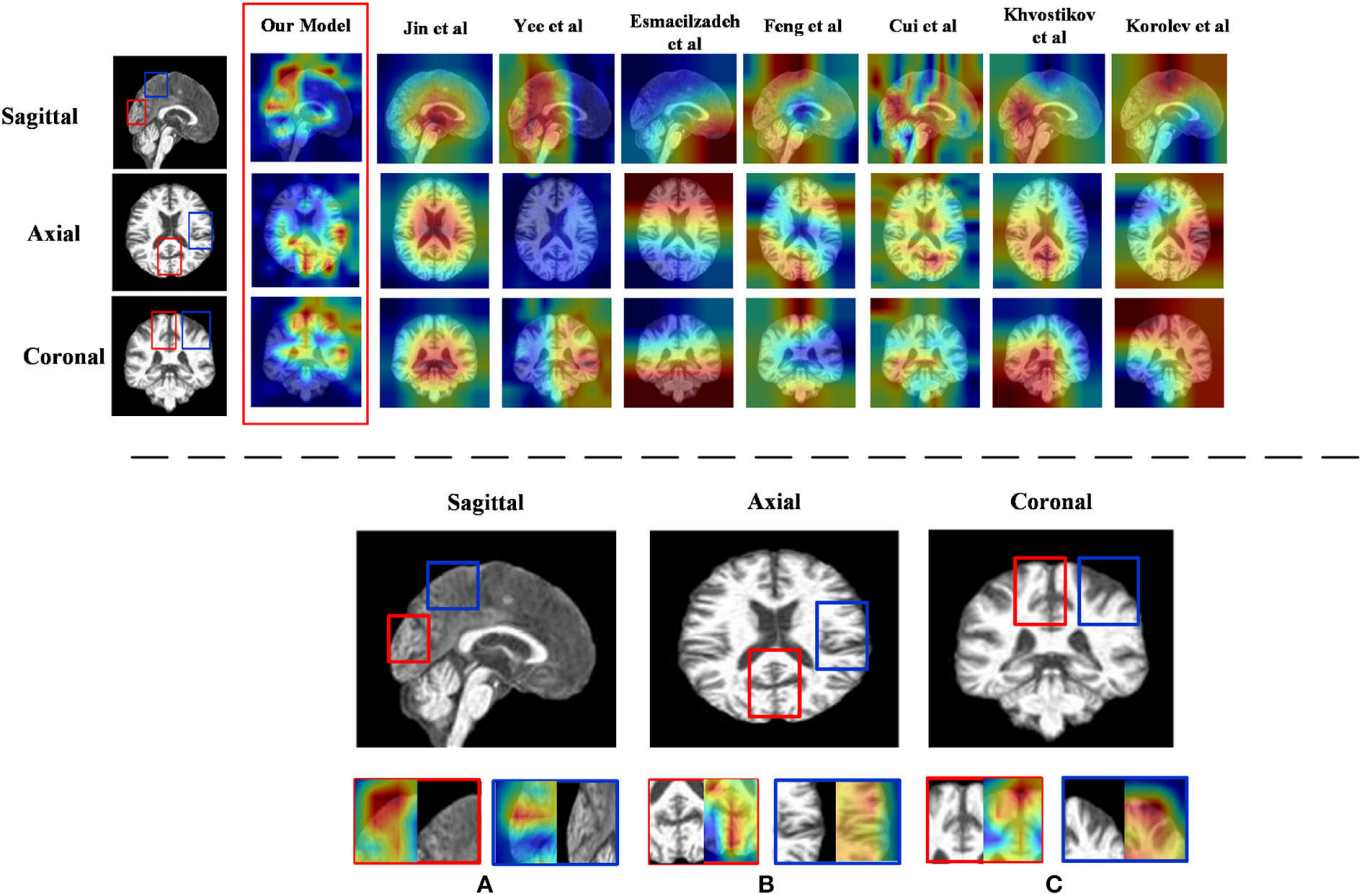
Heatmap visualization of three views of an MRI from ResRepANet and its competing methods(upper panel) and discriminative regions identified by ResRepANet on AD classification (lower panel). In lower panel **(A)** the Sagittal view of MRI and **(B)** the Axial view of MRI while the Coronal view of MRI as **(C)** the heatmaps show the information suggested by attention weights, where the red color denotes the model focus on the regions of locations. The red outlined boxes representing the thalamus belong to the temporal lobe, while the blue outlined boxes represent the cerebral cortex belonging to the parietal lobe.

**Figure 5:**
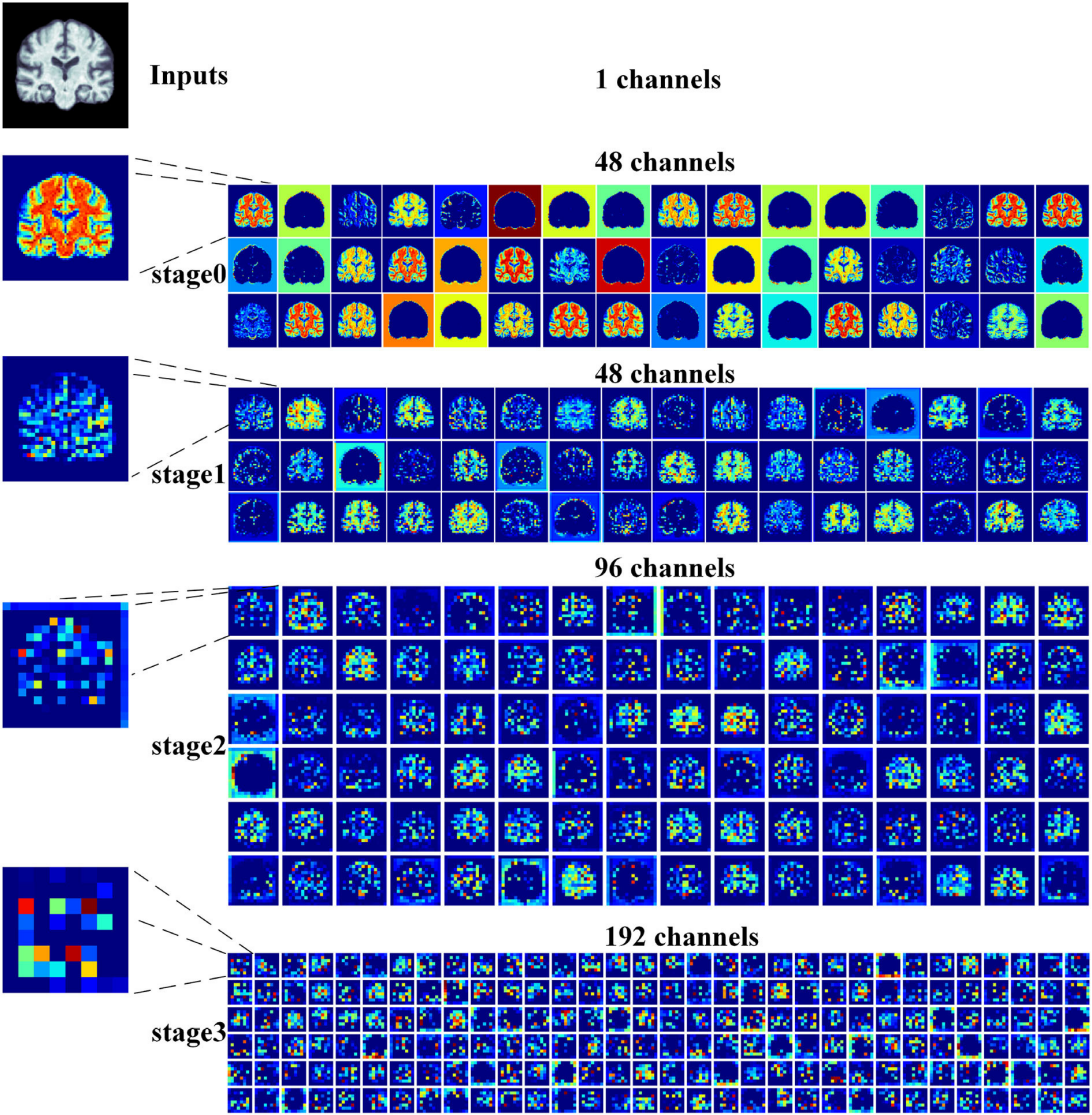
Grad-CAM visualizations of the feature maps produced at different stages by the RepResANet encoder parts. All channel features are derived from one slice of the 3D MRI.

1) *Discriminative region locations*: In order to show discriminative regions directly, we utilized a Grad-CAM (Selvaraju et al., 2019) image to visualize the locations mapped by the convolution feature activations. The discriminative regions are marked from the perspective direction of three views, respectively, and heatmaps were generated to visualize the situation. These heatmaps can show low-to-intermediate implementation difficulties, and their level of interpretability (Ntelios et al., 2012). As can be seen in Figure 4 on the upper panel, the heatmaps generated by our proposed method clearly show that the regions of the thalamus and the regions of the cerebral cortex are mainly covered (Figure 4; lower panel). It can be demonstrated that our proposed methods can distinguish patients with AD precisely by learning the features of the thalamus and cerebral cortex. This is because changes in the thalamus and cerebral cortex are responsible for accessing memory and the capacity for language, which is often one of the first functions to decline noticeably in AD. In contrast, the heatmaps generated by other methods cannot fully cover the efficient locations. For example, Jin et al. (2019) only focused on the thalamus regions, while the attention map of Khvostikov et al. (2018) only distinguished the region of the cerebellum. It is for this reason that the dense features generated by ResRepBlock can better reflect phenotypic lesion characteristics.

2) *Feature map visualization*: In order to observe the process of generating attention maps, we further visualized the coronal view of a feature map in the encoder of our proposed model. According to the structure of ResRepANet, we visualized the process in four stages, as shown in Figure 5. The input sample with one channel is convoluted at stage 0 and generated 48 feature maps with different feature representations. As the network deepens, the feature maps extracted by each ResRepBlock become increasingly abstracted. The visualization process reveals two interesting facets. On one hand, the feature maps generated by stages 0 and 1 can be seen to contain information about contours and the whole of the intracranial region. We can draw a conclusion that our network with its NCSA block can correctly extract representative features. On the other hand, stages 2 and 3 extracted further features from the images generated by stage 1. These modules are responsible for analyzing local features and can be applied initially to produce semantic information.

## 4. Conclusion

In this article, we describe a 3D Residual RepVGG Attention Network (ResRepANet) for the classification of AD to trade off accuracy and speed. The core contribution of this attention network is the NCSA block, which improves the accuracy of the AD classification process. The NCSA block can explore the long-range spatial relevance, and yields a significant performance improvement over an already strong baseline. We also propose a GDMM based on the gradient norm to alleviate the issue of category imbalance. A series of comprehensive experimental tests on an ADNI dataset and an AIBL dataset show that our network achieves a new state-of-the-art performance. However, the approach we propose still has some limitations. For example, the feature maps are more redundant, which is extracted from our proposed ResRepANet (Figure 5; first column). Therefore, we will consider strategies to improve the situation, such as GhostNet. We also intend our proposed ResRepANet to expand its application to other vision-related tasks.

## Data availability statement

The datasets generated during the current study are not publicly available due to the privacy of medical data but are available from the website of Alzheimer's Disease Neuroimaging Initiative (https://adni.loni.usc.edu/) on reasonable request.

## Author contributions

ZC: conceptualization, methodology, and writing-original draft preparation. ZW: writing-reviewing and editing. MZ: data curation. QZ: software. XL: validation. JL: investigation. XS: supervision. All authors contributed to the article and approved the submitted version.

## Funding

This work was sponsored in part by the Key-Area Research and Development Program of Guangdong Province under Grant 2019B010109001, Guangdong Natural Science Foundation under Grant 2020A1515011409, Construction Project of Regional Innovation Capability and Support Guarantee System in Guangdong Province under Grant 2021A1414030004, and Provincial Agricultural Science and Technology Innovation and Extension Project of Guangdong Province under Grant 2022KJ147.

## Conflict of interest

The authors declare that the research was conducted in the absence of any commercial or financial relationships that could be construed as a potential conflict of interest.

## Publisher's note

All claims expressed in this article are solely those of the authors and do not necessarily represent those of their affiliated organizations, or those of the publisher, the editors and the reviewers. Any product that may be evaluated in this article, or claim that may be made by its manufacturer, is not guaranteed or endorsed by the publisher.
